# Supplementary figures and images for: Characterization of CRISPR-Cas Systems in Serratia marcescens Isolated from Rhynchophorus ferrugineus (Olivier, 1790) (Coleoptera: Curculionidae)
Source: Microorganisms. 2019 Sep 19;7(9):368. doi: 10.3390/microorganisms7090368 (PMC6780938; doi:10.3390/microorganisms7090368)

# I-E subtype

CAV1492 (39 DRs)  
CAV1761

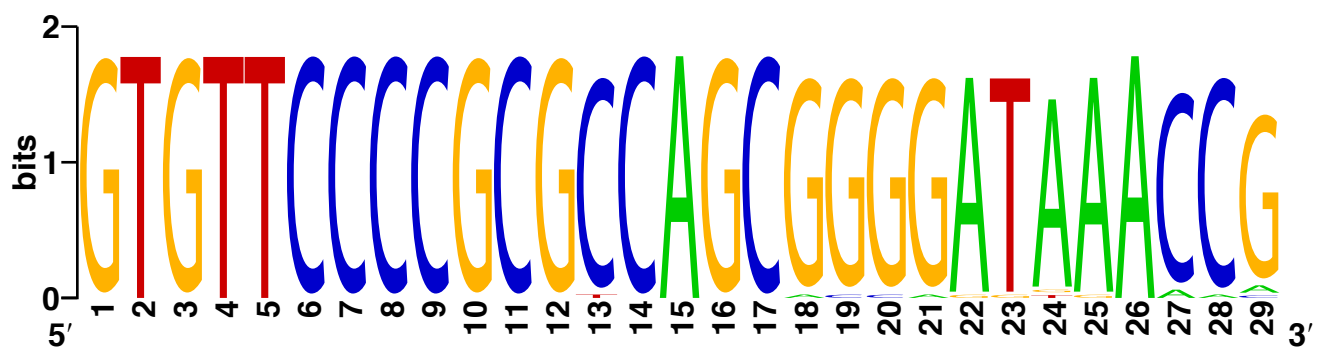

KS10  
EL1 (24 DRs)

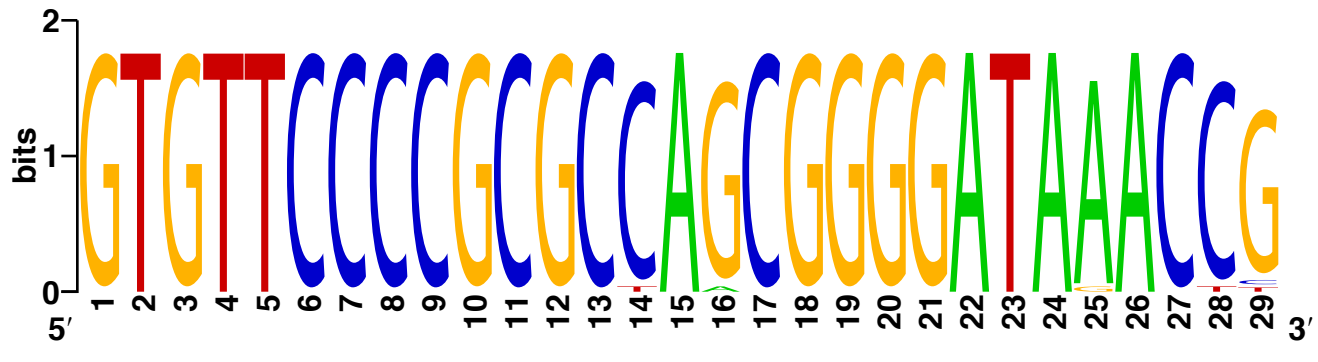

S8 (31 DRs)

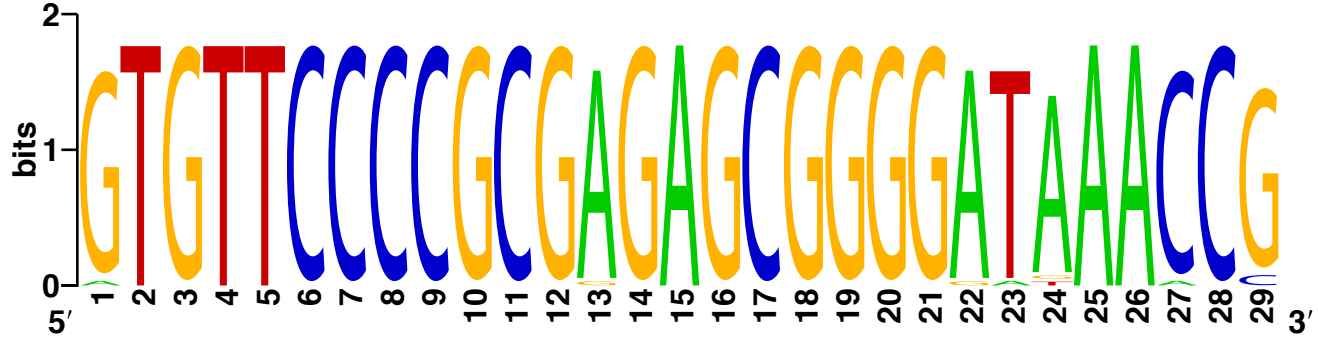

S8 (11 DRs)

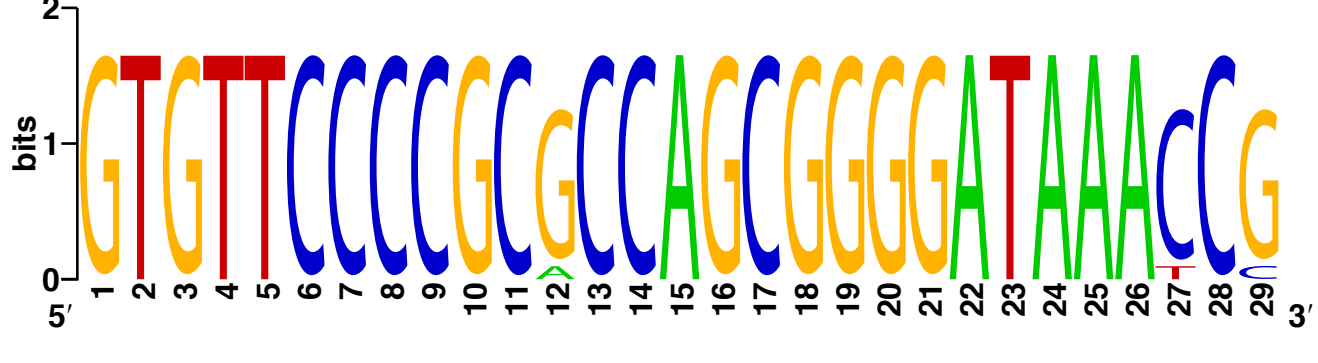

S8 (30 DRs)

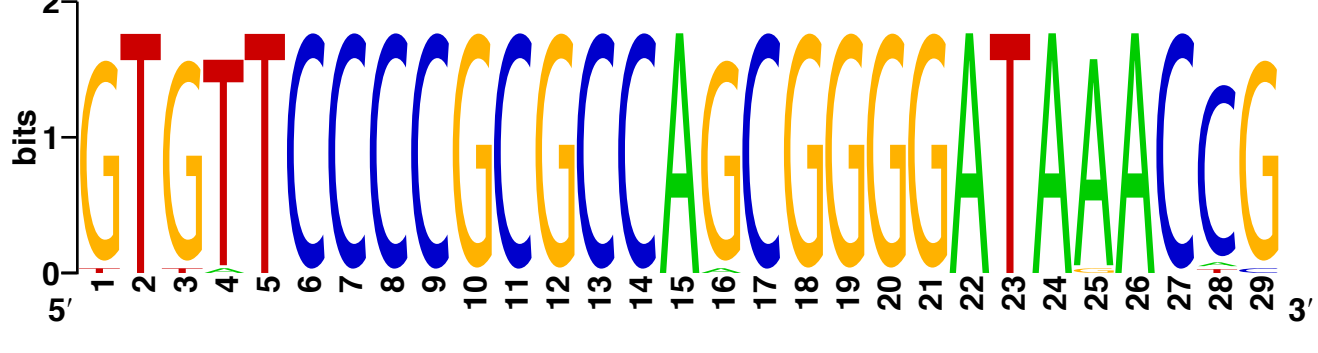

Supplement: Supplementary file 1 [file microorganisms-07-00368-s001.zip › Supplementary materials/Figures S/Figure S1.pdf]

# I-F subtype

12TM (26 DRs)

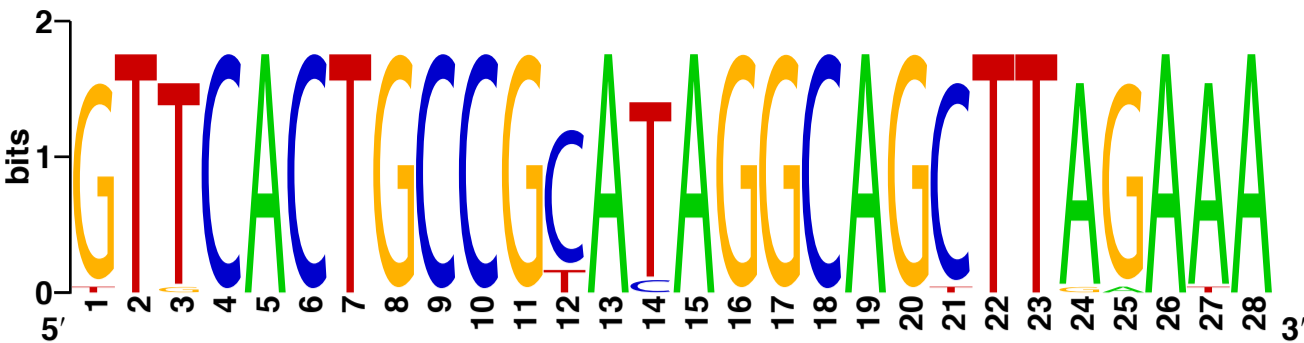

12TM (28 DRs)

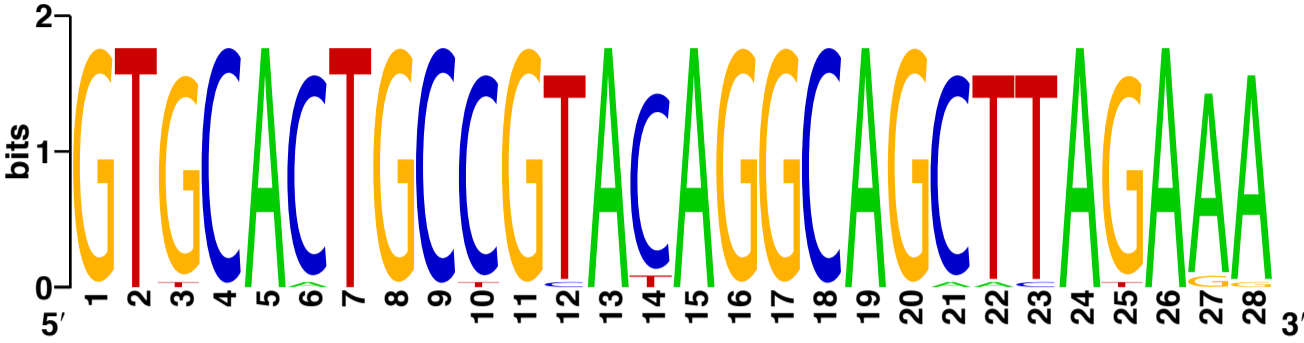

B3R3 (14 DRs)

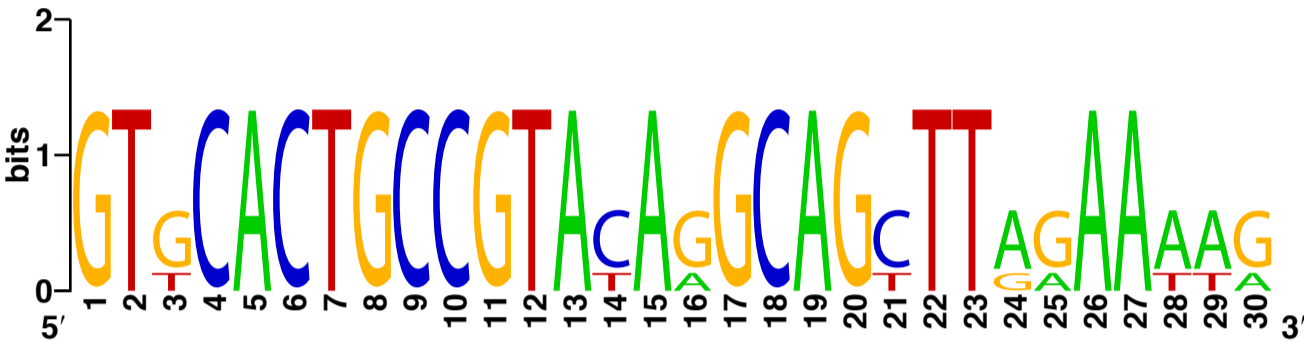

N4-5 (9 DRs)

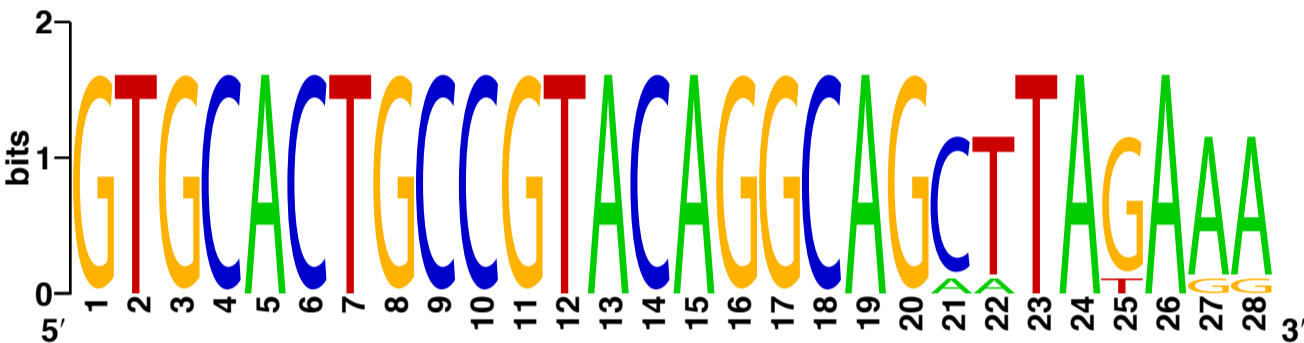

N4-5 (18 DRs)

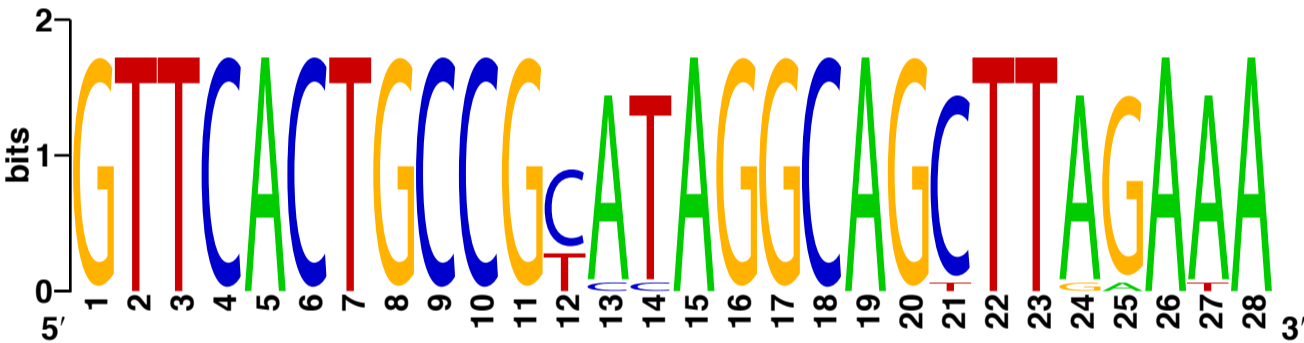

PWN146 (7 DRs)

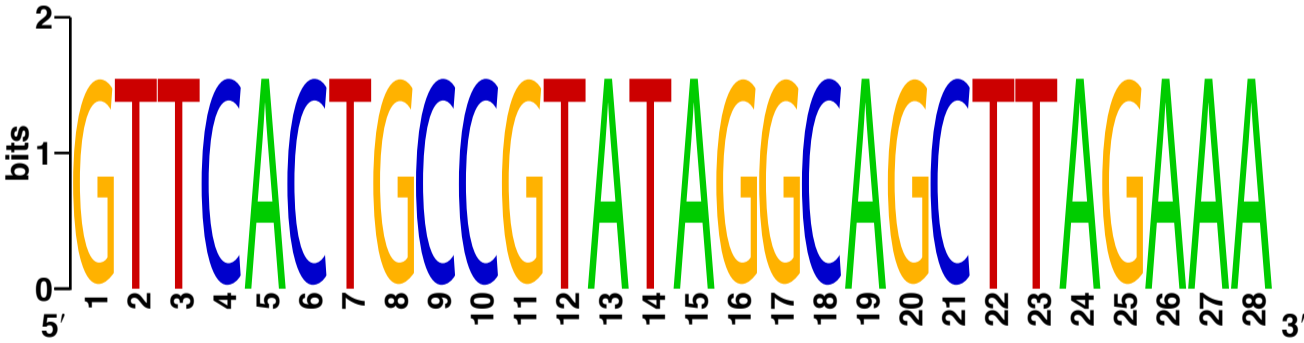

PWN146 (47 DRs)

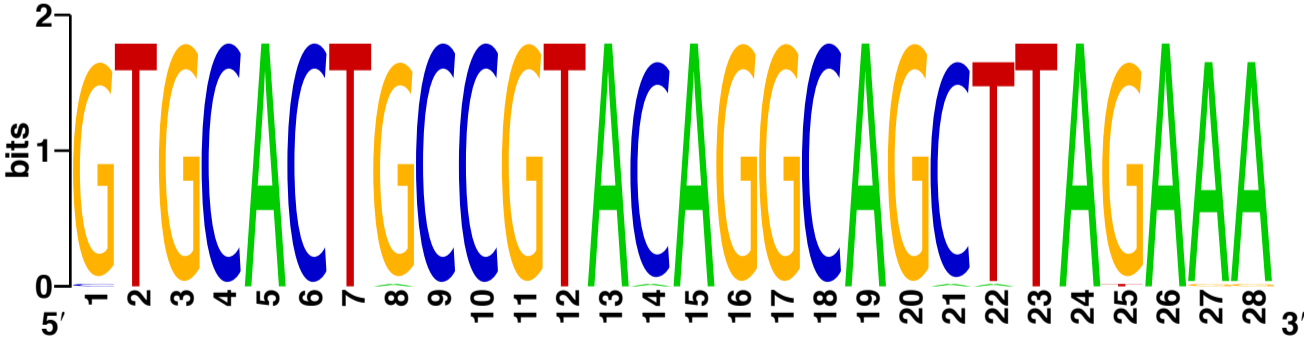

S5 (4 DRs)

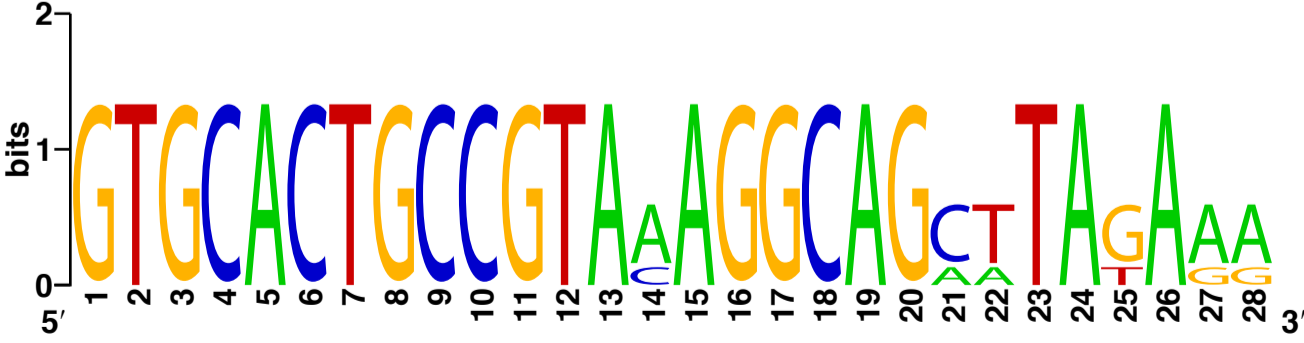

S5 (51 DRs)

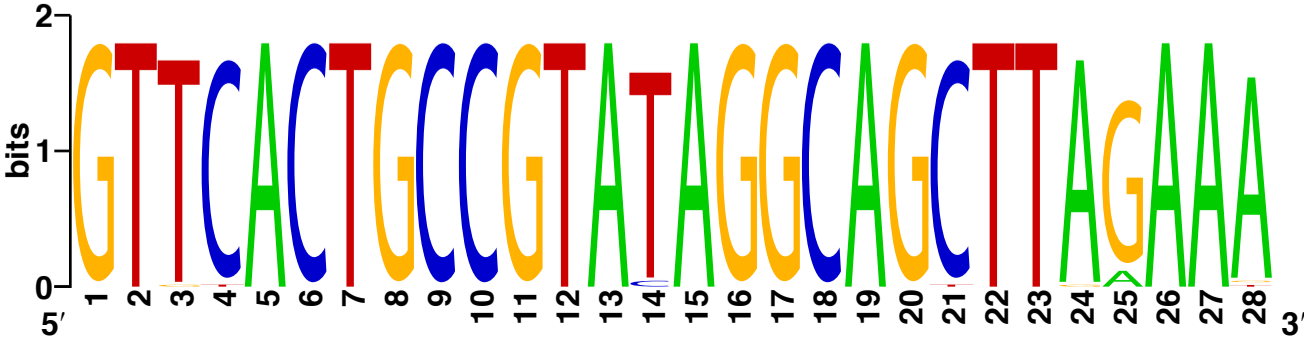

Supplement: Supplementary file 1 [file microorganisms-07-00368-s001.zip › Supplementary materials/Figures S/Figure S2.pdf]

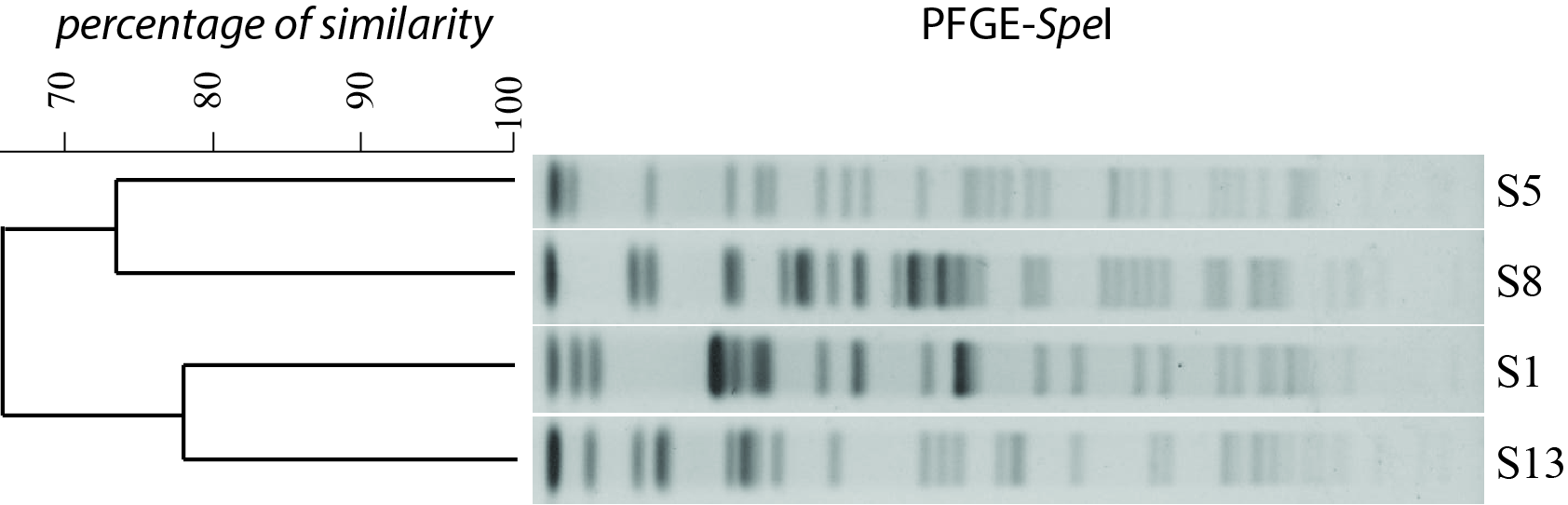

Supplement: Supplementary file 1 [file microorganisms-07-00368-s001.zip › Supplementary materials/Figures S/Figure S4.tif]
